# Impact of Temperature on the Biochemical Potential of Five Newly Isolated Strains of Microalgae Cultured in a Stirred Tank Reactor

**DOI:** 10.3390/microorganisms13051155

**Published:** 2025-05-18

**Authors:** Panagiotis Dritsas, George Aggelis

**Affiliations:** Department of Biology, School of Natural Sciences, University of Patras, 26500 Patras, Greece; dritsas.p@ac.upatras.gr

**Keywords:** *Picochlorum costavermella*, *Picochlorum oklahomense*, *Microchloropsis gaditana*, *Nephroselmis pyriformis*, high added-value metabolites

## Abstract

The microalgal strains *Picochlorum costavermella* VAS2.5, *Picochlorum oklahomense* SAG4.4, *Picochlorum oklahomense* PAT3.2B, *Microchloropsis gaditana* VON5.3, and *Nephroselmis pyriformis* PAT2.7 were cultured in a Stirred Tank Reactor at 25 °C or 20 °C in modified artificial seawater and their biotechnological potential was assessed. VAS2.5, VON5.3, and PAT2.7 were high in biomass production at both temperatures (i.e., 438.8–671.3 mg/L and 418.4–546.7 mg/L at 25 °C and 20 °C, respectively), though *P. oklahomense* strains grew only at 25 °C. The highest lipid percentage was recorded for the cultures of VAS2.5 (19.3 ± 0.7%) and VON5.3 (16.4 ± 1.5%) at 25 °C, notably rich in ^Δ5,8,11,14,17^C20:5, while PAT2.7 proved a major producer of ^Δ9^C16:1. The predominant lipid fraction was glycolipids and sphingolipids (41.3–57.4%) for VAS2.5, PAT2.7 at 25 °C and VON5.3 at 20 °C and neutral lipids (55.6–63.5%) in the other cultures, indicating the different effect of temperature on lipid synthesis of the various microalgae. Additionally, almost all strains stood out for their high protein content, exceeding 50% in the culture of PAT3.2B, but polysaccharide and pigment content were not high. The biochemical profiles of the isolates showcased their suitability for use primarily as feed additives in the aquaculture sector.

## 1. Introduction

Microalgae are photosynthetic microorganisms with a global distribution, found not only in all types of water systems but in terrestrial ecosystems and the atmosphere as well [1,2,3]. Besides their obvious high ecological value, microalgae, through the conversion of solar energy and CO_2_, biosynthesize cellular materials of high nutritional interest (e.g., lipids, proteins, carbohydrates, pigments, etc.) for aquatic and terrestrial organisms in significant quantities [4,5]. Typically, green microalgae, rather than other groups of photosynthetic microorganisms, are considered as a superior oil source compared to conventional plants for a number of reasons. The major ones are related to their higher growth rates and their ability to grow under a wider range of pH and temperature values and valorize CO_2_ in a more efficient way comparing to plants [6,7,8].

More importantly, some microalgal species, e.g., representatives of the genera *Picochlorum* and *Microchloropsis*, are of additional interest due to their ability to synthesize polyunsaturated fatty acids (PUFAs), such as the omega-3 fatty acids α-linolenic (^Δ9,12,15^C18:3, ALA), eicosapentaenoic (^Δ5,8,11,14,17^C20:5, EPA), and docosahexaenoic (^Δ4,7,10,13,16,19^C22:6, DHA). Such fatty acids are sought after by the food, feed, and pharmaceutical industries because of their proven beneficial effects against cardiovascular and neurodegenerative diseases, various forms of cancer, etc. [9,10,11,12]. On the other hand, microalgal lipids that contain more saturated fatty acids, like those that belong to the *Nephroselmis* genus, are used as feedstock for biodiesel production, because they are resistant to oxidation and yield more energy during combustion [13,14,15].

In general, the major advantage of microalgal oils against the fish oils, which are the main source of omega-3 fatty acids, is that they are free of undesirable odors and pollutants such as heavy metals and microplastics [16,17]. Additionally, the extensive use of microalgal oils will contribute decisively to the context of controlling and reducing intensive fishing globally. After all, microalgae farming incurs low production costs and, thus, can offer economic benefits by reducing dependency on imported fish oil in countries of need [18,19]. Yet even though >50,000 species of microalgae are described, only a few are cultivated on a large scale due to their high production costs [20,21,22].

Therefore, the need to use new strains with high efficiency rates when used in large-scale applications is of outmost importance. However, a crucial challenge that is associated with the yield of lipid production is the design of proper culture conditions. Environmental factors, such as temperature, irradiance, and nutrient availability majorly affect lipid content and fatty acid composition in microalgae cells [23,24,25]. For instance, at low temperatures, one might expect increased synthesis of unsaturated fatty acids, including PUFAs, as cells need to maintain the fluidity of their membranes, increasing their PUFA content. Evidently, in low temperatures, higher yields of these molecules of high biotechnological significance can be achieved [26,27].

The aim of the present study was to investigate the effect of temperature on the physiology and high added-value metabolite synthesis capability of five strains of microalgae, previously isolated and characterized with molecular techniques [13], that belong to genera *Picochlorum*, *Microchloropsis*, and *Nephroselmis*, due to the attractive features of representatives of the aforementioned genera and the relatively limited available literature for some of these strains [13,28,29,30,31,32], when cultured in a Stirred Tank Reactor (STR). One of the main reasons for using these strains to serve the cultures in the current investigation relates to the relatively limited literature available on most of these strains and the lack of data on the potential utilization of such microorganisms on a commercial scale. Even though the main focus was on lipid production, the fatty acid composition of total lipids and their lipid fractions, and the content of these strains in proteins, polysaccharides, and pigments were also analyzed. As a result of the aforementioned analyses, the biotechnological potential of the used strains when cultured in the STR was assessed.

## 2. Materials and Methods

### 2.1. Biological Material and Culture Conditions

The microalgal strains *Picochlorum costavermella* VAS2.5, *Picochlorum oklahomense* SAG4.4, *Picochlorum oklahomense* PAT3.2B, *Microchloropsis (Nannochloropsis) gaditana* VON5.3, and *Nephroselmis pyriformis* PAT2.7, which were previously isolated from coastal areas of the Ionian Sea of Greece and identified by the use of molecular techniques [13], were used as biological material.

The strains were maintained in 0.25 L Erlenmeyer flasks containing 0.05 L of modified Artificial Sea Water (mASW) and were regularly sub-cultured. The composition of mASW is presented in Table 1. The flasks containing mASW were sterilized at 121 °C for 20 min, inoculated with 10^5^ cells/mL of a fresh inoculum, derived from a 7-day pre-culture grown in sterilized mASW, and incubated at T = 25 ± 1 °C. Illumination was constant with an intensity of 300 μmol/m^2^∙s provided by linear fluorescent day light tubes (T5, 8 W, 6500 K). Before sterilization, the pH of the growth medium was adjusted to 8.5 ± 0.5 by adding 2.5 M NaOH (Sigma-Aldrich) solution and using an Orion 420A digital pHmeter (Thermo Scientific, Osterode, Germany) equipped with a glass electrode (HANNA Instruments, Athens, Greece). The pH of mASW was also measured after sterilization.

**Figure 1:**
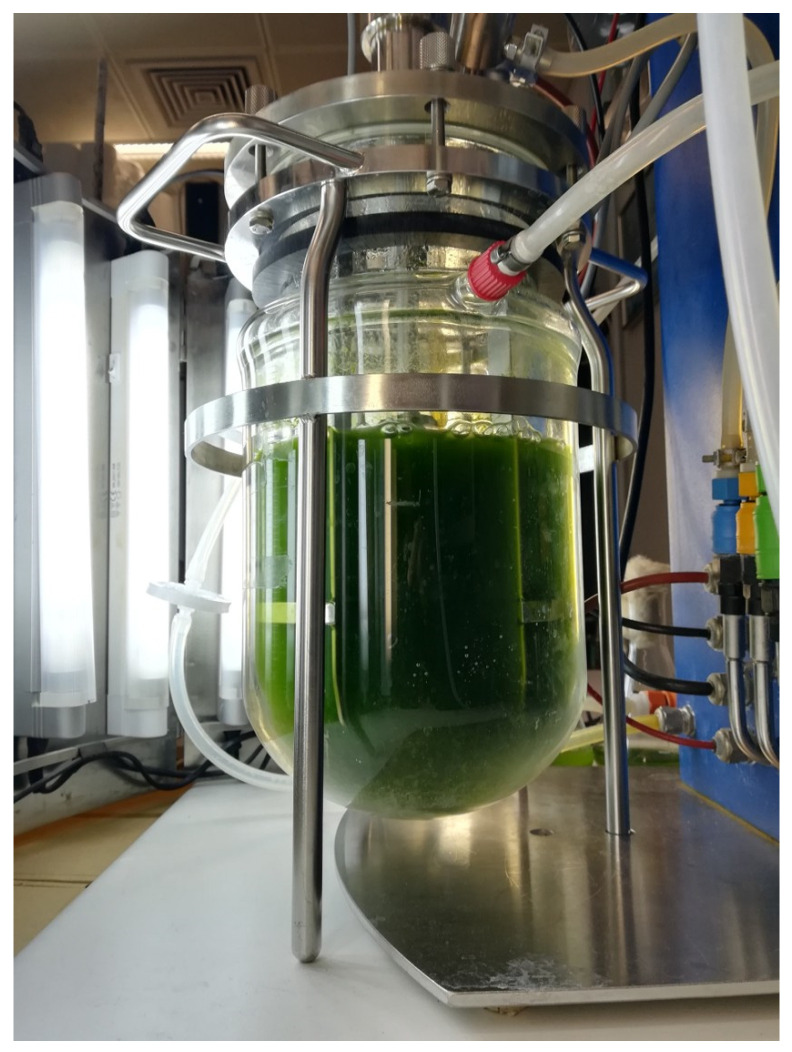
Cultures of selected microalgae in a Stirred Tank Reactor (STR) (V_w_ = 2 L). Culture conditions: modified artificial seawater (mASW), pH: 8.5 ± 0.3, temperature: 25 ± 1 °C or 20 ± 1 °C, photoperiod: 24:0, light intensity: 1071 μmol/m^2^∙s, agitation: 100 rpm, aeration: 0.5 vvm.

The microalgal strains were cultured in monoalgal but non-axenic batch cultures for approximately 250 h in a closed system bioreactor, type Ralf Plus-System from Bioengineering (Wald, Switzerland), which is a Stirred Tank Reactor (STR), with a capacity of 3.7 L and a working volume (V_w_) = 2 L. The STR was converted into a light bioreactor after 8 fluorescent lamps (T5, 8 W, 6500 K) were placed circumferentially to the culture vessel, providing continuous illumination of 1071 μmol/m^2^∙s intensity (Figure 1). Prior to culture initiation, the culture vessel was cleaned with 70% ethanol (vol/vol), approximately 1.8 L of sterile mASW was added, and inoculation was performed with approximately 0.2 L of pre-cultured microalgae so that the initial cell density was 1.5 ∙ 10^6^ cells/mL. The cultures were maintained at T = 25 ± 1 °C or T = 20 ± 1 °C under 100 rpm agitation and natural air was supplied at a rate of 0.5 vvm, which was passed through a Whatman-type bacteriological filter with a pore size of 0.2 μm. Both the selected temperatures correspond to the sea temperatures from which the original samples were collected. The initial pH was 8.5 ± 0.3 and was maintained at this value by the addition of 2.5 M HCl (Sigma-Aldrich) automatically added to the medium. At the end of each culture, the biomass was collected for quantification and further chemical analyses. Cultures were carried out in duplicate. The monitoring of the culture conditions and the physicochemical parameters of the system was possible through a computer equipped with Wonderware InTouch 9.5.001 software (Bioengineering, Wald, Switzerland).

### 2.2. Cell Growth and Biomass Determination

Daily cell counting in a Neubauer improved cell counting chamber (Poly-Optik, Bad Blankenburg, Germany) was performed, similar to other studies [33,34], so as to assess microalgae growth. In brief, 1 mL of microalgae culture was subtracted from the culture vessel daily, diluted properly, and in each slot of the Neubauer chamber, 10 μL of microalgae sample was placed. Then, under an optical microscope Carl Zeiss Axiostar (GmBH, Gottingen, Germany), at total magnification of 400×, cell density was calculated following the equation provided below (Equation (1)):

*N_av._ per square* ∙ *DF* (if any) ∙ 25 ∙ 10^4^ cells/mL (1)

where *N_av._ per square* is the average number of cells per square of the Neubauer chamber and *DF* is the dilution factor.

The cell counts were expressed as microalgal cell density (cells/mL). In addition, the integrated version of Verhulst’s model (i.e., Sigmoidal Logistic function) (Equation (2)) was applied in order to estimate the parameters of growth:

*N_t_* = *N_max_*/(1 + *b* ∙ e^1/^*^μ^^∙t^*) (2)

where *N* represents the number of cells/mL∙10^6^ at time *t*, *b* is a positive constant equal to *(N_max_ − N*_0_*)/N*_0_ (where *N*_0_ is the initial number of cells/mL∙10^6^), *μ* is the specific growth rate (1/d), and *N_max_* is the carrying capacity of the system. The parameter values were determined by fitting Equation (1) to the experimental data, using the Levenberg–Marquardt method for parameter value optimization. The minimization of the residual root means square error between the experimental and model-predicted data, quantified by the values of the coefficient of determination R^2^, was used as a criterion for parameter optimization.

Biomass determination was held by gravimetric determination of dry biomass. Specifically, prior to the gravimetric determination, harvesting of the microalgal cells by centrifugation at 7455× *g* for 10 min at 4 °C (NÜVE NF 800R, Ankara, Turkey) occurred. Then, washing of the cells with deionized water and centrifugation under the aforementioned conditions occurred in duplicate and then the biomass was collected and dried at 80 °C until constant weight and expressed as g/L.

### 2.3. Lipid Extraction and Purification

Total lipids from the microalgal cells were extracted in chloroform (PENTA)/methanol (Fisher Chemical, Hampton, NY, USA) 2:1 vol/vol, in accordance with a modified version of the Folch et al. (1957) [35] method [36]. Then, the extracts were filtered through Whatman No. 1 paper, washed with a 0.88% (wt/vol) KCl (Merck) solution in order to remove non-lipid components (i.e., lipoproteins, pigments), and dried over anhydrous Na_2_SO_4_ (Sigma-Aldrich). Consequently, solvent evaporation under vacuum, using a Rotavapor R-210 evaporator (BUCHI, Flawil, Switzerland), occurred and the total cellular lipids were gravimetrically determined and expressed as the percentage of lipids in the dry biomass (L/x%, wt/wt).

### 2.4. Lipid Fractionation

A column (25 × 100 mm) containing 1 g silicic acid (Fluka), which was activated by heating at 80 °C overnight, was used for the fractionation of approximately 100 mg of microalgal lipids which were priorly dissolved in 1 mL chloroform. The column was washed successively with 100 mL dichloromethane (Sigma-Aldrich) to obtain neutral lipids (N), 100 mL acetone (Fluka) to obtain glycolipids plus sphingolipids (G + S), and 50 mL methanol (Sigma-Aldrich) to obtain phospholipids (P) [33]. Following the evaporation under vacuum of the respective solvent of each fraction, all fractions (N, G + S, and P) were quantified gravimetrically and expressed as a percentage of total lipids.

### 2.5. Fatty Acid Composition of Cellular Lipids

The identification of total lipids and their lipid fraction composition in fatty acids was made attainable after their conversion into their fatty acid methyl-esters (FAMEs) and analysis with Gas Chromatography (GC). Specifically, an Agilent 7890A GC device (Agilent Technologies, Shanghai, China) equipped with a flame ionization detector at 280 °C and an HP-88 (J&W Scientific, Folsom, CA, USA) column (60 m × 0.32 mm) was used. The carrier gas was helium, at a flow rate 1 mL/min, and the analysis was run at 200 °C. Peaks of FAMEs were identified through comparison to authentic standards.

### 2.6. Polysaccharide Determination

Approximately 20 mg of fat-free biomass (x_f_) was hydrolyzed with 5 mL HCl (Sigma-Aldrich) 2.5 M at 100 °C for 60 min. When the hydrolysate was cooled at room temperature, it was neutralized with 2.5 M KOH (Sigma-Aldrich) and filtered through Whatman No. 1 paper so as to remove cell debris. The reducing sugars, expressed as glucose, were determined in the solution in accordance with the DNS method [37]. Intracellular polysaccharides, including storage and structural polysaccharides, were expressed (as glucose equivalents) as a percentage of dry biomass (S/x%, wt/wt).

### 2.7. Protein Determination

Total cellular protein was determined using approximately 10 mg of x_f_ following the biuret method, as described elsewhere [33]. Cellular protein was expressed (as albumin equivalents) as a percentage of dry biomass (P/x%, wt/wt).

### 2.8. Pigment Estimation

Approximately 0.5 g of wet biomass was solubilized with 10 mL of ethanol 95%, vol/vol (Fisher Chemical) solved with Milli Q water (Honeywell, Charlotte, NC, USA). The mixture was centrifuged (Hettich Mikro 200R, Föhrenstr, Germany) at 13,300× *g* for 15 min at 4 °C. Then, 0.5 mL of the supernatant was collected and mixed with 4.5 mL of the ethanol solution [38]. The resulting solution was spectrophotometrically analyzed for Chlorophyll-a, Chlorophyll-b, and carotenoids in a quartz cuvette (1 cm^2^), while ethanol solvent was used as a blank.

The Equations (3)–(5) used for the pigment quantification (in µg/mL) were the following [22]:

*C_a_* = (13.36∙*A_664_*) − (5.19∙*A_649_*) (3)

*C_b_* = (27.43∙*A_649_*) − (8.12∙*A_664_*) (4)

*C_x+c_* = ((1000∙*A_470_*) − (2.13∙*C_a_*) − (97.63∙*C_b_*))/209 (5)

where *A* represents Absorbance and *C_a_*, *C_b_*, and *C_x+c_* stand for Chlorophyll-a, Chlorophyll-b, and carotenoids, respectively. Pigments, evaluated as Total Chlorophylls (TCh) and Total Carotenoids (TC), were expressed as percentage in the dry biomass (TCh/x%, wt/wt, and TC/x%, wt/wt).

### 2.9. Data Treatment and Statistical Analysis

The graphing and analysis software OriginPro 2021 9.8.0.200 ^®^, 1991–2020 (OriginLab Corp., Northampton, MA, USA) was used for the treatment of the experimental data from microalgal cultures and growth kinetics.

## 3. Results

### 3.1. Cell Growth and Biomass Production

**Figure 2:**
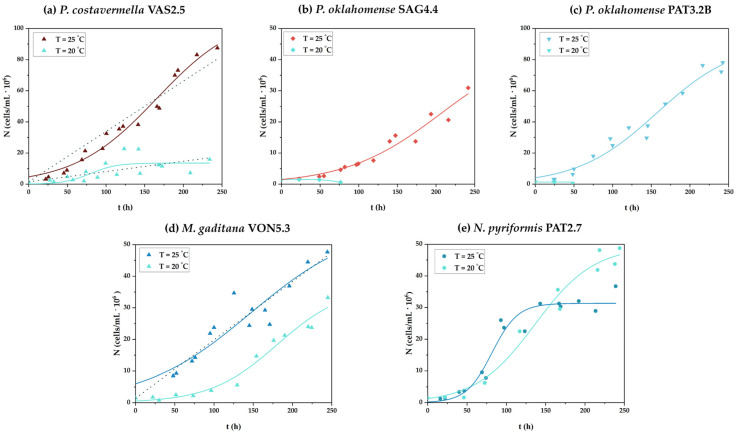
Growth curves of (**a**) *Picochlorum costavermella* VAS2.5, (**b**) *Picochlorum oklahomense* SAG4.4, (**c**) *Picochlorum oklahomense* PAT3.2B, (**d**) *Microchloropsis gaditana* VON5.3, and (**e**) *Nephroselmis pyriformis* PAT2.7 grown in mASW in a 3.7 L (V_w_ = 2 L) capacity Stirred Tank Reactor (STR) (two biological replicates). Each point is an average of two measurements and the curves were obtained by fitting the experimental data to the integrated form of Verhulst’s model. The dotted lines refer to the application of the linear growth model to the experimental results. Culture conditions: modified artificial seawater (mASW), pH = 8.5 ± 0.3; temperature: 25 ± 1 °C or 20 ± 1 °C; photoperiod: 24:0; light intensity: 1071 μmol/m^2^∙s; agitation rate: 100 rpm; aeration: 0.5 vvm.

The growth curves of all strains under the examined growth conditions are collectively depicted in Figure 2, while the results regarding growth parameters are compiled in Table 2.

The strain *P. costavermella* VAS2.5 grew satisfactorily at both incubation temperatures tested, especially at T = 25 °C (Figure 2a), leading to higher biomass production as well (x = 671.3 ± 17.2 mg/L with *p_x_* = 67.1 ± 1.7 mg/L∙d and *μ* = 0.45 ± 0.05 1/d), compared to 418.4 ± 17.7 mg/L of the other culture condition (Table 2). It is worth mentioning that due to the similarity of the conceivable curve that could express the trend of the cytometry points with a straight line when this strain was cultured at T = 25 °C, the linear model was also applied to the data, showing a very good fit (R^2^ = 0.98) (see respective dοtted line plot in Figure 2a). Similar observations were made regarding the culture at T = 20 °C (see respective dotted line plot in Figure 2a) as well as for the other two strains that belong to the *Picochlorum* genus and were used in the current study. Intriguingly, though, the other two *Picochlorum* isolates, namely, *P. oklahomense* SAG4.4 and *P. oklahomense* PAT3.2B, showed growth capacity only when incubated at T = 25 °C (Figure 2b,c). Under these culture conditions, both strains reached similar biomass production levels. Specifically, *P. oklahomense* SAG4.4 biomass production reached 224.8 ± 5.9 mg/L (*p_x_* = 22.5 ± 0.7 mg/L∙d and *μ* = 0.39 ± 0.08 1/d) and *P. oklahomense* PAT3.2B produced 242.7 ± 13.6 mg/L (*p_x_* = 24.3 ± 1.4 mg/ L∙d and *μ* = 0.47 ± 0.07 1/d) (Table 2).

Regarding the cultures of *M. gaditana* VON5.3, this strain grew satisfactorily at both examined temperatures, but clearly more efficiently when incubated at T = 25 °C (Figure 2d). As mentioned before for *P. costavermella* VAS2.5 cultured at T = 25 °C, the growth curve of the *M. gaditana* VON5.3 culture at the same temperature was also well-fitted to the linear model as shown by the dotted line (Figure 2d) (R^2^ = 0.90 and R^2^ = 0.88 for linear model and the integrated version of Verhulst’s model, respectively). As regards the biomass production, *M. gaditana* VON5.3 gained practically the same level of biomass at both incubation temperatures (Table 2). In particular, it was x = 527.4 ± 15.0 mg/L (*p_x_* = 52.7 ± 1.5 mg/L∙d and *μ* = 0.35 ± 0.11 1/d) and x = 509.0 ± 77.8 mg/L (*p_x_* = 50.9 ± 7.8 mg/L∙d and *μ* = 0.56 ± 0.12 1/d) for the culture at T = 25 °C and T = 20 °C, respectively.

The strain *N. pyriformis* PAT2.7 also grew satisfactorily at both incubation temperatures (Figure 2e). Interestingly, in this set of experiments, the biomass produced was lower when the microalga grew at T = 25 °C, i.e., x = 438.8 ± 89.3 mg/L (with *p_x_* = 43.9 ± 8.9 mg/L d and *μ* = 1.47 ± 0.34 1/d) compared to x = 546.7 ± 1.2 mg/L (with *p_x_* = 54.7 ± 0.1 mg/L∙d and *μ* = 0.64 ± 0.12 1/d) at T = 20 °C (Table 2).

### 3.2. Accumulation of Storage Materials

The results of storage material accumulation levels at the end of each culture performed are compiled in Table 2.

Regarding *P. costavermella* VAS2.5, it was observed that the lipid content was clearly higher at T = 25 °C (L/x% = 19.3 ± 0.7%, wt/wt), whereas at T = 20 °C, the lipid yield was relatively low (L/x% = 5.2 ± 0.3%, wt/wt). The other two *Picochlorum* strains, which were able to grow only at T = 25 °C, synthesized fewer lipids, i.e., L/x% = 4.1 ± 0.4%, wt/wt and L/x% = 11.5 ± 0.3%, wt/wt, for *P. oklahomense* SAG4.4 and *P. oklahomense* PAT3.2B, respectively (Table 2). Additionally, the percentages (%, wt/wt) of lipid fractions (N—neutral lipids, G + S, glycolipids, and sphingolipids, P—phospholipids) to total lipids (TLs) are also presented in Table 2. Herein, though, such investigation occurred only for *P. costavermella* VAS2.5 cultured at T = 25 °C, as it was not possible to investigate the contribution of individual lipid fractions to total lipids of the biomass collected from the lower temperature, due to the small amount of lipids produced. Likewise, the aforementioned results apply to the two *P. oklahomense* strains as well. In this respect, the predominant lipid fraction of *P. costavermella* VAS2.5 was that of G + S (57.4 ± 5.4%, wt/wt), followed by the N fraction (29.6 ± 3.4%, wt/wt) and P (13.1 ± 2.0%, wt/wt). *M. gaditana* VON5.3 produced biomass in which the reserve lipid content was slightly higher at T = 25 °C (L/x% = 16.4 ± 2.1%, wt/wt) compared to the culture at T = 20 °C (L/x% = 13.8 ± 0.0%, wt/wt). The dominant lipid fraction was that of N (55.6 ± 1.6%, wt/wt), followed by the G + S fraction (24.7 ± 1.4%, wt/wt) and P (19.8 ± 0.3%, wt/wt) when the microalgae was grown at T = 25 °C. On the contrary, the highest percentage was recorded for the G + S fraction (41, 3 ± 2.0%, wt/wt) after culture at T = 20 °C, with P also following at high percentages (36.3 ± 2.1%, wt/wt), and finally, the N fraction (22.5 ± 0.2%, wt/wt) (Table 2). Lastly, as regards the biomass content of *N. pyriformis* PAT2.7 to reserve lipids in the two incubation conditions, a higher percentage was observed in the culture at T = 20 °C (L/x% = 9.3 ± 6.1%, wt/wt) compared to that at T = 25 °C (L/x% = 3.3 ± 1.7%, wt/wt) (Table 2). When this strain was grown at T = 25 °C, the highest percentage belonged to the lipid fraction G + S (48.6 ± 5.0%, wt/wt) which was marginally higher than the N fraction (45.8 ± 4.3%, wt/wt), and the last one was the P fraction (6.0 ± 1.0%, wt/wt). In culture at 20 °C, there was a clear dominance of the N fraction (63.5 ± 6.0%, wt/wt), followed by G + S (34.8 ± 4.8%, wt/wt) and P (2.3 ± 1.2%, wt/wt) (Table 2).

Regarding the ability of *P. costavermella* VAS2.5 to store polysaccharides and proteins under the examined culture conditions, a significant difference was recorded in the accumulation of polysaccharides (S/x% = 13.5 ± 0.8 and S/x% = 6.7 ± 0.1, for cultures at T = 25 °C and T = 20 °C, respectively), but not in protein content (P/x% ranged in the 25.0–26.7%, wt/wt range) (Table 2). Similar levels of polysaccharides and protein synthesis were recorded for *P. oklahomense* SAG4.4 (S/x% = 11.3 ± 0.4%, wt/wt and P/x% = 28.8 ± 1.0%, wt/wt) cultured at T = 25 °C (Table 2). On the other hand, despite polysaccharide accumulation being similar to the above for *P. oklahomense* PAT3.2B (i.e., S/x% = 11.2 ± 1.2%, wt/wt), the exhibited protein content of the biomass was significantly higher (P/x% = 50.6 ± 3.0%, wt/wt) (Table 2). Regarding the ability of *M. gaditana* VON5.3 to store polysaccharides and proteins under the two culture conditions, similar percentages with little variation were also recorded (S/x% = 8.5–9.4% and P/x% = 16.4–17.5%) (Table 2). In the same manner, small variations were recorded in the case of *N. pyriformis* PAT2.7, though the recorded protein content was high (S/x% = 8.0–10.7% and P/x% = 41.0–45.2%) (Table 2).

Finally, the values of total chlorophyll and carotenoids in dry biomass were recorded (Table 2). The obtained results presented similar levels of chlorophyll biosynthesis (the percentage of which ranged between 2.1 and 2.6%, wt/wt) and slightly higher levels of carotenoids when *P. costavermella* was grown at T = 20 °C (0.8% at t = 20 °C versus 0.2%, wt/wt at T = 25 °C). On the contrary, the cultures of both *P. oklahomense* strains at T = 25 °C led to higher total chlorophyll (TCh/x% = 4.9 ± 0.1%, wt/wt and TCh/x% = 6.1 ± 0.7%, wt/wt, for SAG4.4 and PAT3.2B, respectively) and carotenoid (TC/x% = 0.8 ± 0.0%, wt/wt and TC/x% = 1.1 ± 0.1%, wt/wt, for SAG4.4 and PAT3.2B, respectively) content of the cells. Respective analyses on *M. gaditana* VON5.3 biomass showed higher levels of chlorophyll biosynthesis when the microalga was grown at T = 20 °C (TCh/x = 3.2% vs. 1.4%) and similar rates of carotenoid biosynthesis when grown at T = 25 °C (i.e., TCh/x = 0.3–0.9%, wt/wt and TC/x = 0.1–0.3%, wt/wt). Finally, total chlorophyll and carotenoid contents were at low levels in both culture conditions tested for *N. pyriformis* PAT2.7 (in the range of TCh/x% = 1.0–1.7%, wt/wt, and TC/x% = 0.3%, wt/wt).

### 3.3. Fatty Acid Composition of Total Lipids and Lipid Fractions

The composition of total lipids and lipid fractions in fatty acids at the end of the cultures under both culture conditions are listed in Table 3, Table 4 and Table 5.

Regarding the culture of *P. costavermella* VAS2.5 at T = 25 °C, it is observed that in TLs the predominant fatty acids are C16:0 (29.0 ± 0.2%, wt/wt) and ^Δ9^C16:1 (28.6 ± 0.3%, wt/wt), followed by ^Δ5,8,11,14,17^C20:5 presenting in a significant proportion (17.0 ± 0.2%, wt/wt). The N lipid fraction showed a similar fatty acid composition to that of the TLs, except that the proportion of ^Δ5,8,11,14,17^C20:5 in it was significantly lower (5.4 ± 0.1%, wt/wt). The percentages of fatty acids within the G lipid fraction were similar to those of TLs. With regard to the P fraction, a decrease in the percentage of C16:0 and ^Δ9^C16:1 was observed, which was accompanied by a significant increase in the percentage of ^Δ5,8,11,14,17^C20:5 to 30.3 ± 4.9% (wt/wt), compared to TLs. The three aforementioned fatty acids were also dominant in the TL cultures at T = 20 °C; however, in this case, limited biosynthesis of C16:0 was observed (14.3 ± 0.6%, wt/wt), in contrast to ^Δ5,8,11,14,17^C20:5, whose concentration was higher (35.9 ± 0.7%, wt/wt), compared to that of lipids produced at T = 25 °C.

Regarding the fatty acid composition of total lipids produced by *P. oklahomense* SAG4.4, the predominant fatty acid is the rare fatty acid ^Δ6,9,12,15^C18:4, (18.9 ± 3.6%, wt/wt), followed at similar levels by ^Δ9^C18:1 (18.4 ± 2.4%, wt/wt), while C16:0 (12.2 ± 2.1%, wt/wt) came in third place (Table 3). Also, Table 3 shows that the fatty acid ^Δ9,12,15^C18:3 was also present at remarkable levels (6.3 ± 0.6%, wt/wt). On the other hand, in the case of *P. oklahomense* PAT3.2B, the fatty acid detected in the highest percentage was ^Δ9,12^C18:2 (25.3 ± 0.0%, wt/wt), followed by the fatty acids ^Δ9,12,15^C18:3 (17.9 ± 1.0%, wt/wt), C16:0 (15.2 ± 0.1%, wt/wt), and ^Δ9^C18:1 (14.4 ± 0.8%, wt/wt) (Table 3). Other fatty acids detected, but at significantly lower percentages, were ^Δ9^C14:1 (9.3 ± 0.3, wt/wt), C18:0 (6.4 ± 0.7, wt/wt), ^Δ9^C16:1 (4.4 ± 0.4, wt/wt), and C14:0 (2.3 ± 0.2, wt/wt) (Table 3).

In the case of *M. gaditana* VON5.3, it was observed for the culture at T = 25 °C that in TLs the dominant fatty acids were ^Δ9^C16:1 (30.5 ± 0.8%, wt/wt) and C16:0 (24.1 ± 1.3%, wt/wt), followed by ^Δ5,8,11,14,17^C20:5 (19.9 ± 0.1%, wt/wt) (Table 4). The lipid N fraction showed a similar pattern to that of TLs, but contained ^Δ5,8,11,14,17^C20:5 at a significantly lower percentage (10.4 ± 1.9%, wt/wt). The lipid fraction G showed a similar fatty acid composition to TLs, while the P fraction showed a small percentage of ^Δ9^C16:1 and a large percentage for C16:0 (36.3 ± 1.0%, wt/wt) and C18:0 (17.6 ± 0.9%, wt/wt) fatty acids, while ^Δ5,8,11,14,17^C20:5 was detected at the levels of the N fraction. The three fatty acids that dominated at 25 °C were also those with the highest percentages when *M. gaditana* was grown at 20 °C, and at similar levels to those determined in the culture incubated at T = 25 °C (Table 4). As shown in Table 4, there is a correspondence with the 25 °C growth case in terms of the fatty acid composition of the lipid fractions, with the only difference being the high percentage of ^Δ5,8,11,14,17^C20:5 (29.5 ± 3.3%, wt/wt) in the lipid fraction of P.

Lastly, the predominant fatty acids of *N. pyriformis* PAT2.7 were ^Δ9^C16:1 (40.1 ± 0.1%, wt/wt) and C14:0 (31.0 ± 0.8%, wt/wt) in TLs when this strain was grown at T = 25 °C, and the N and G lipid fractions showed a similar pattern to that of TLs (Table 5). However, the presence of C14:0 at lower amounts (15.5 ± 0.7%, wt/wt) was recorded for the P lipid fraction and a remarkable percentage for ^Δ9^C18:1 (19.4 ± 2.3%, wt/wt), compared to TLs and the two aforementioned lipid fractions. When *N. pyriformis* was grown at T = 20 °C, a similar pattern to the 25 °C growth was observed in terms of the composition and fatty acid content of total lipids and their lipid fractions (Table 5).

## 4. Discussion

Microalgae are used in a plethora of biotechnological applications and several species are being used to produce several tons of biomass and metabolites of high biotechnological value [4,5,21]. Concurrently, microalgae are an important biological material for basic research [39]. The main cellular components that determine the caloric value and quality of biomass of photosynthetic microorganisms intended for feeding terrestrial and aquatic organisms are lipids, proteins, and polysaccharides [3,4,21]. Some species are of additional interest because of their ability to synthesize lipids that contain PUFAs in high concentrations. PUFAs are quite popular in the food, feed, and pharmaceutical industries due to their proven beneficial effects against various threats to human and animal health [9,10,11]. On the other hand, species which contain more saturated fatty acids can be used in biodiesel production [7,21,40].

All isolates used in the present study were previously characterized biochemically and cultured under laboratory conditions, exhibiting satisfactory yields regarding growth, biomass, and high added-value metabolite production [13]. Herein, special emphasis was given to the ability of the selected microalgal strains to accumulate significant amounts of lipids rich in PUFAs (i.e., *Picochlora* and *M. gaditana* VON5.3), or more saturated lipids suitable for biodiesel (i.e., *N. pyriformis* PAT2.7) and, secondly, to polysaccharide, protein, and pigment production. The predominant reserve material in all microalgae was proteins, followed by lipids and polysaccharides. Another factor for the selection of these strains was the limited literature available on species of the genera *Picochlorum* and *Nephroselmis*; thus, the opportunity to offer useful data regarding exploring the exploitation potential of such microorganisms has arisen.

The STR was chosen for the cultures performed since it offers better control on culture conditions, e.g., regarding temperature, aeration, agitation, etc. Another crucial factor was the ability of considerable high illumination supply (i.e., 1071 μmol/m^2^∙s). Moreover, Sorokin and Krauss (1958) [41], in early studies, after examination of several strains of the genus *Chlorella* (related to *Picochlorum* as both genera belong to the order Chlorellales), under light intensities of about 300 μmol/m^2^∙s and higher, concluded that in green microalgae the maximum growth rate is achieved under continuous illumination at intensities up to saturation of the photosynthetic capacity of the microalgae. The aforementioned conclusions are also confirmed by the later study of Sukenin and Carmeli (1989) [42] who, apart from biomass production, linked the synthesis of lipids and polysaccharides to the intensity of light provided to a *Nannochloropsis* sp. strain grown in F/2 nutrient medium on turbidostat at three different light intensities (35 μmol/m^2^∙s, 290 μmol/m^2^∙s, 550 μmol/m^2^∙s). De la Vega et al. (2011) [43], at supplied light intensities of 100 and 1,200 μE/m^2^∙s, recorded a small increase in the specific growth rate (from 0.031 1/h to 0.034 1/h) of *Picochlorum* sp. HM1, concluding that its growth is nearly saturated at relatively low light intensities, while no photoinhibition was observed for light intensities of 1,200 μE/m^2^∙s. In any case, it has to be noted that the findings of this research may suggest the existence of two limiting factors for growth, which were probably the dissolved CO_2_ concentration, as atmospheric air, containing 0.03% CO_2_, was provided to the cultures, and lighting, which is often a limiting factor in high volume cultures.

Another parameter to be considered regarding the light energy supply, which can potentially influence biomass growth, is ‘mutual shading’ [44,45,46]. Specifically, an increase in cell concentration can be associated with an increase in the ‘mutual shading’ caused by preventing light from penetrating deeper into the culture. This phenomenon is also observed in the natural environment of microalgae, which can cause shading of either cells of the same species or cells of other species, affecting the balance of the ecosystem. In this respect, the lateral light supply in the agitated cultures carried out in the STR contributed positively to a better uptake of light energy by the cells. In this context, a safe strategy to increase the synthesis of pigments, especially carotenoids, is considered to be the provision of high light intensity or a gradual increase in temperature in the culture environment, as both are parameters that stress the cells and activate the photoprotection mechanisms of microalgae [47]. However, in the present study, regardless of culture conditions, a generally similar, but not significantly high, production of chlorophylls was observed when comparing to other studies [29,48,49,50,51,52]. Of note, the pigment content of *M. gaditana* VON5.3 was higher than other strains, e.g., *Nannochloropsis* sp., used in their study by Magpusao et al. (2024) [53]. In any case, the synthesis of chlorophyll-a was higher than chlorophyll-b (i.e., results are given as total chlorophyll values) in all measurements, which is in agreement with the findings other studies [48,49]. However, since all the examined strains are known producers of carotenoids, rich in lutein and zeaxanthin beta-carotene, siphonaxanthin, neoxanthin, etc. [20,43,53,54,55,56], which are of high biotechnological value due to their proven antioxidant and anti-inflammatory activity, culture conditions should be modified when the desired product is these molecules.

Herein, the conclusion drawn from the cultures in the STR of the three *Picochlora* strains was that they all exhibited relatively high specific growth rates and gained satisfying amounts of biomass. The results were comparable or better to the cultures previously performed in Erlenmeyer flasks [13]. Specifically, *P. costavermella* VAS2.5 produced biomass that exceeded 670 mg/L at T = 25 °C, i.e., almost twice the size compared to the experiments in Erlenmeyer flasks [13], while even at T = 20 °C, biomass production reached 418 mg/L. On the other hand, both *P. oklahomense* strains, which were able to grow only at T = 25 °C, reached similar biomass values between them and when compared to their respective cultures in Erlenmeyer flasks [13]. The preference of *Picochlora* for relatively high temperatures has been recorded elsewhere as well [47,57]; thus, the response of the *P. oklahomense* strains came as no big surprise. In any case, taking into consideration that the cultures of this investigation lasted half the time of cultures in Erlenmeyer flasks, it is highlighted that, for these strains, light supply played a key role in increased productivities. Notably, the potential of *Picochlora* to gain high biomass yields is confirmed by other studies as well. For example, Zhu and Dunford (2013) [58] who cultured a *P. oklahomense* strain in a 2 L bioreactor at medium F/2 and even applied a 12:12 photoperiod, recorded biomass production at a significantly higher level (x = 2.1 g/L). Intriguingly, strains such as *Picochlorum* sp. HM1 [43] and *Picochlorum celeri* [59], which present short generation times and similar growth rates with the strains used herein, are directly competitive to commercial strains belonging to genera such as *Dunaliella* and *Nannochloropsis*. However, despite the aforementioned studies, it must be taken into account that, to date, *Picochlora* have not been used extensively in commercial/industrial scale applications. Specifically, to the best of our knowledge, the only study involving the culture of a *Picochlorum oculatum* strain at pilot (V_w_ = 150 L) and commercial scale (V_w_ = 2000 L) in an open horizontal bioreactor outdoors belongs to Dogaris et al. (2019) [60]. The researchers concluded that this strain can be a good candidate for use in the biodiesel production industry. From the above study, it can be concluded that strains of the genus *Picochlorum* could pose as attractive candidates for large-scale applications.

*M. gaditana* VON5.3 grown in an STR showed a higher specific growth rate and higher biomass production, similarly to *Picochlora* strains, compared to its culture in Erlenmeyer flasks [13]. However, it is worth mentioning that in this study a similar amount of biomass was also produced when this strain was cultured at T = 20 °C, demonstrating its very good adaptation even in lower temperature environments. In agreement with the observations on the positive effect of incubation under high light intensity, another study on *N. salina* also shows a positive correlation of the increase in biomass production with the intensity of the light energy provided [61]. On the other hand, *N. salina* grown in mASW in what resembles an open-pond system reactor at 120 μE/m^2^∙s, produced biomass of equivalent amount to that of *M. gaditana* VON5.3 [33]. Simionato et al. (2011) [62] supported that within a certain light energy threshold little change in biomass production occurs, while only under extreme light intensities can significant changes in biomass production be observed. However, when *N. oculata* was grown in 2 L containers on growth medium F/2, with a light output of 56 ± 4 μmol/m^2^∙s and photoperiod of 12:12, it produced biomass that reached 1.2 g/L, though in a culture that lasted almost as long [58].

*N. pyriformis* PAT2.7 showed high specific rate values. Even though *N. pyriformis* PAT2.7 gained more biomass when grown in Erlenmeyer flasks [13], significant biomass production was recorded in STR at both examined temperatures, as well, especially taking into consideration that these cultures lasted half the time compared to the ones in Erlenmeyer flasks. Noteworthily, in this case, growth appeared to be more efficient when *N. pyriformis* was cultured at T = 20 °C. The results of the research by Hotos and Avramidou (2021) [63] seem to verify the above observation, since they recorded satisfactory growth and remarkable biomass production from another *Nephroselmis* strain when cultured at T = 20–21.5 °C. Nevertheless, it should be mentioned that other studies recorded the production of equivalent or even more biomass when representatives of the genus were grown at higher temperatures (e.g., 25–27 °C) [29,40,64]. From the above observations, it can be concluded that species of this genus exhibit plasticity responsive to different temperature values, with the optimal growth temperature varying between species or even strains. This observation suggests that the intensity of the light energy provided, although clearly high, had neither a positive nor a negative effect on the growth of the microalgae. However, it should be mentioned that the intershading from the very day of the cultures played an important role since the cultures showed a dark green color, indicating the insufficient availability of the supplied light in the inner part of the culture volume.

With regard to lipid biosynthesis, the lowest yields for *P. costavermella* VAS2.5 were observed when grown at 20 °C, which can be attributed to the environmental stress conditions and the need to maintain cell homeostasis. All other values regarding *Picochlora* strains used in this study were comparable to their respective cultures in Erlenmeyer flasks [13]. Lipid accumulation of the *P. oklahomense* strain used by Zhu and Dunford (2013) [58] was similar to the one presented by *P. costavermella* VAS2.5 at T = 25 °C. However, Tran et al. (2014) [65] characterized a strain of the genus that gained up to L/x% = 48.6% (wt/wt) under stress conditions and low temperature (T = 15 °C), while its growth rate was not severely affected. The aforementioned observations confirm the plasticity of the representatives of this genus. Higher lipid content was observed for *M. gaditana* VON5.3 compared to when it was grown in Erlenmeyer flasks (i.e., L/x% = 9.3 ± 3.0%, wt/wt) [13] at both culture temperatures, while no significant effect of temperature was recorded for the amount of lipids synthesized between the two cultures. The increased yields compared to the cultures in Erlenmeyer flasks can be accounted for by the high light energy supplied to the cultures grown in this type of bioreactor, in accordance with the findings of Mohammady (2014) [61]. In any case, lipid content of this strain was comparable to that of the commercial strain *Nannochloropsis* sp. of Reed Mariculture (USA), which though it exceeded 17% (wt/wt), was significantly lower than *N. oceanica* F&M-M24, which approached 30% (wt/wt) [53,66]. With *N. pyriformis* PAT2.7 cultures, lipid synthesis was low at T = 25 °C, while it was considered adequate under culture at T = 20 °C. The available literature on the ability of *Nephroselmis* to accumulate lipids is quite limited, yet the results of the present study are comparable to those of another *Nephroselmis* strain (L/x% = 10.5%, wt/wt), which was grown for about one week in F nutrient medium at 25 °C, with a salinity of 28‰ and a light intensity of 80–100 μmol/m^2^∙s [67]. However, the *Nephroselmis* sp. that Hotos et al. (2023) [68] cultured in Walne medium accumulated lipids at 15%, wt/wt. Even higher lipid accumulation was exhibited by *Nephroselmis* sp. KGE1, also grown on BBM, with L/x% = 33.0 ± 0.06% (wt/wt) [64]. BBM, taking into account its composition, is a growth medium more suitable for freshwater microalgae, highlighting the adaptability of *Nephroselmis* in various aquatic systems, which has been confirmed elsewhere (e.g., Hotos et al. (2020) [69]). Yet, it could also be noted that BBM contains KNO_3_ = 0.25 g/L as a nitrogen source, compared to 1 g/L contained in mASW, implying that in *Nephroselmis* lipid production is be significantly influenced by the availability of nitrogen rather than the temperature in the growth environment.

Regarding the content of total lipids in their lipid fractions, it must be taken into consideration that a common feature for a plethora of microalgae is the dominance of the G + S fraction over total lipids, as has been reported for strains of various genera, like *Chlorella*, *Nannochloropsis*, and *Tetraselmis* [33,34,49,70]. The above is answered by the fact that G + S are the main components (in percentages up to 80–90%) of lipids of chloroplast and thylakoid membranes [71,72,73,74]. Glycolipids of microalgae are considered an important source of omega-3 fatty acids, while they can also play the role of storage material [75,76]. Sphingolipids are a diverse group of membrane lipids that were highly conserved during evolution and play an important role in cell communication with its environment and other cells as well as in several important cell functions, such as the control of cell division [77,78]. The P fraction also belongs to the predominant structural lipids in microalgae, as phospholipids are a key component of all cell membranes. Biosynthesis of the polar lipids is strongly affected by several factors, such as the composition of the nutrient medium, the provided light intensity and photoperiod, and the growth phase of the culture [79]. However, one of the most influential factors is environmental temperature. In low temperature, the presence of saturated lipids in cell membranes reduces their fluidity and negatively affects cellular metabolism. Consequently, an increased synthesis of unsaturated fatty acids is expected, which could increase survival under such adverse conditions [26,27].

The main lipid fraction, for *P. costavermella* VAS2.5 at T = 25 °C, the sole case that was possible to perform such analyses among the cultures performed using the *Picochlora* strains, was that of G + S, followed by N lipid fraction and then P. In the same manner, from the cultures of *M. gaditana* VON5.3, the fraction G + S was also expected to be the dominant one, as documented elsewhere [33,34]. However, when this strain was cultured at T = 25 °C, the N fraction dominated; albeit, this could be attributed to the high light intensity [80]. *N. gaditana* 1049 was also shown to synthesize mainly N lipids, though after the 16th day of cultivation [81]. The authors interpreted this phenomenon as a consequence of a shift in the biochemical pathways of lipid biosynthesis of an aged culture from chloroplasts and other cell membranes mainly towards N lipid production. Another study denoted growth of *Nannochloropsis* sp. SCSIO-45217 under phosphorus-limiting conditions as favorable for synthesis of N lipids [82]. In contrast, when was *M. gaditana* VON5.3 cultured at T = 20 °C, it presented high G + S content, aligning with the aforementioned observations regarding the need of cells to remodel their cell wall and photosynthetic system in response to low temperature. Overall, since G + S and P are more abundant in PUFAs, the *M. gaditana* strain VON5.3, especially under relatively low temperatures, appears to be a particularly attractive candidate for production of PUFAs in large-scale applications. In contrast to the other microalgae, *N. pyriformis* PAT2.7 proved to be a major producer of N lipids and, secondarily, of G + S. The very high content in N lipid reserves is another indication of the ability of this microalga to survive at T = 20 °C.

In any case, the examined microalgal strains presented a notable fatty acid composition. *P. costavermella* VAS2.5 synthesized ^Δ5,8,11,14,17^C20:5 at high concentrations, exceeding the P fraction, where the PUFAs are biosynthesized, 30% (wt/wt) at 25 °C, or even 35% (wt/wt) at 20 °C in total lipids. The ^Δ5,8,11,14,17^C20:5 content of this strain can explain the very low percentages or even the occasional absence of ^Δ9,12,15^C18:3 and ^Δ6,9,12,15^C18:4. According to the mechanisms described by Bellou et al. (2014) [4] and Jesionowska et al. (2023) [83], very long chain PUFAs (VLC-PUFAs) are synthesized by the catalytic action of desaturases and elongases on ^Δ9^C18:1 via the ω-3 and/or ω-6 pathways. In brief, C16:0 is initially synthesized within chloroplasts through the enzymatic action of the fatty acid synthase (FAS) complex and is then converted to C18:0 by the addition of two carbon atoms derived from acetyl-CoA. The latter one is then desaturated to ^Δ9^C18:1 which in its activated form is transferred to the endoplasmic reticulum where it is further desaturated by Δ12 and Δ15 desaturases and produces ^Δ9,12^C18:2 and ^Δ9,12,15^C18:3. The last ones serve as precursors for the ω-6 and ω-3 pathways, respectively, for the biosynthesis of VLC-PUFA. In the ω-3 biosynthetic pathway, ^Δ9,12,15^C18:3 is converted to ^Δ6,9,12,15^C18:4, ^Δ8,11,14,17^C20:4, and then to ^Δ5,8,11,14,17^C20:5 by desaturase Δ6, elongase Δ6, and desaturase Δ5, respectively. ^Δ5,8,11,14,17^C20:5 is a PUFA of high biotechnological significance, since it is a biosynthetic precursor of prostaglandins-3 which inhibit platelet aggregation, thromboxane-3, and eicosanoids [83]. The presence of this fatty acid appears to have been recorded in only one other representative of the genus, though at much lower levels [65].

The main fatty acids produced by *M. gaditana* VON5.3 were the same as when grown in Erlenmeyer flasks [13], namely, ^Δ9^C16:1 and ^Δ5,8,11,14,17^C20:5. Total lipids and lipid fractions of *M. gaditana* VON5.3 were generally similar in composition, and the ^Δ5,8,11,14,17^C20:5 was detected in high proportions, in polar lipids. At the same time, ^Δ9^C16:1 was also high in polar lipids, especially at T = 20 °C. A similar pattern in total lipids and their lipid fractions was also recorded for other *Microchloropsis* and *Nannochloropsis* strains elsewhere [33,34,53]. More importantly, *M. gaditana* VON5.3 showed a significantly higher ^Δ5,8,11,14,17^C20:5 content compared to the nine *Nannochloropsis* and *Microchloropsis* strains used by Ma et al. (2014) [84]. In their study, the most productive strain in terms of ^Δ5,8,11,14,17^C20:5 did not exceed 13% (wt/wt) on total lipids. Also, *Nannochloropsis* sp. SCSIO-45217 presented significantly lower ^Δ5,8,11,14,17^C20:5 content (i.e., 2.1–7.3%, wt/wt) than *M. gaditana* VON5.3 when grown under phosphorus-limiting conditions (at concentrations of 4–40 mg/L), albeit it synthesized ^Δ9^C16:1 in significant amounts (35.2–37.1%, wt/wt) [82]. Even lower was the lipid content of the aforementioned fatty acids in the case of *N. oceanica* F&M-M24 [66]. The above observations suggest the ability and suitability of *M. gaditana* VON5.3 as a source of lipid production of high nutritional and medicinal value, especially cultured at T = 20 °C, which presented further enhanced yields in PUFA synthesis.

*N. pyriformis* PAT2.7 synthesized mainly saturated fatty acids and an absence of PUFAs was recorded, with the exception of traces of ^Δ6,9,12^C18:3 detected in some cases. These features highlight *N. pyriformis* PAT2.7 as a remarkable candidate for the biodiesel production industry. The predominant fatty acid in all cases was ^Δ9^C16:1 at high percentages of total lipids. Moreover, ^Δ9^C16:1 occurred in similar proportions in the N and G + S lipid fractions, while its proportion was reduced to about half in the P fraction. The significant production of ^Δ9^C16:1 is considered to be of outstanding importance and possibly opens up new prospects for the exploitation of this microalga, since ^Δ9^C16:1 is attributed with anti-inflammatory properties and the ability to reprogram the intestinal microflora. Other strains of the genus synthesized much smaller amounts of ^Δ9^C16:1 (3.9–6.1%, wt/wt) [40,67] but also linolenic acids (^Δ6,9,12^C18:3 and ^Δ9,12,15^C18:3), which in total approached 15% (wt/wt) of total lipids. Even higher yields were recorded in other studies regarding ^Δ6,9,12^C18:3 content of total lipids (i.e., 37.7–56.4%) [51,64]. The above studies further support the intra-species variability and the need to select the proper species/strain in correspondence to the desired final product.

Regarding polysaccharide accumulation in *Picochlora* strains, their content was almost double in comparison with their respective cultures in Erlenmeyer flasks [13], yet not in considerable amounts. Similarly, *M. gaditana* VON5.3 synthesized polysaccharides at slightly higher levels compared to Dritsas et al. (2023) [13]’s respective culture and at comparable amounts to the *N. salina* used by Bellou and Aggelis (2012) [33]. However, strains that belong to *Microchloropsis* and *Nannochloropsis* can accumulate polysaccharides in higher levels (i.e., 20–25%, wt/wt) [34,53,66]. *N. pyriformis* PAT2.7 produced polysaccharides in the same range of values with other strains of the genus [13,51,67]. According to Bellou and Aggelis (2012) [33], the amount of intracellular reserve materials is linked with the growth phase of microalgae, while the authors also considered reduced photosynthesis and CO_2_ availability as crucial factors. Specifically, they concluded that the passage of light through the culture and CO_2_ captured at high cell densities can lead to not covering the metabolic needs of the cells in the culture. Consequently, the cells meet the need for energy and metabolic precursors at the expense of the reserve materials. In addition, it is worth noting that Mastropetros et al. (2023) [52] suggest high salinity and light intensity as environmental stressors for increased polysaccharide production in *Nephroselmis*.

Regarding the protein content of *Picochlorum* strains, high accumulation rates were recorded independent of culture temperature, especially for *P. oklahomense* PAT3.2B, in which protein content exceeding 50% (wt/wt). The protein synthesis was comparable to the cultures of these strains in Erlenmeyer flasks [13]. The above did not come as a surprise, as generally the ability of *Picochlora* to accumulate proteins at high levels has been documented elsewhere [48,49,58,85]. Interestingly, the existence of amino acid profiles of high commercial and biotechnological interest has been reported for other strains of the genus [56,65,86]. When *M. gaditana* VON5.3 was grown in the STR, the protein content of the biomass was about half compared to the experiments in Erlenmeyer flasks [13], which may possibly be explained by the increased metabolic activity of the cells and the high biomass production in a relatively short incubation period. In each case, the protein content under these conditions was comparable to that of other *Microchloropsis* or *Nannochloropsis* strains [33,34,58], while temperature practically did not affect protein synthesis. On the other hand, a particularly high content was observed in *N. pyriformis* PAT2.7 cultures in both temperatures, slightly higher when this microalga was cultured at T = 20 °C. Protein synthesis was comparable to the cultures of this strain previously performed in Erlenmeyer flasks [13]. The high protein content of *N. pyriformis* PAT2.7 grown at 20 °C demonstrates its good adaptation to these conditions and suggests the suitability of the strain for large-scale outdoor cultivation where temperatures are low during the winter months. Moreover, the very high protein content of *Nephroselmis* highlights it as capable of being utilized in the diet of fish (directly or indirectly) or even humans. It appears that under conditions of environmental stress the microalgae channel energy towards energy conservation and/or growth rather than towards protein synthesis.

Taking into consideration the above observations, it reasonably follows the deduction and the ambition that this investigation can contribute to the design of the proper culture conditions, taking into account parameters such as temperature, adapted to the species or even strains of the same species, with the aim to optimize yields of the desired products. This would offer the possibility and the prospect for the development of various applications of microalgae, both in the aquaculture sector and in the production of high added-value products through technologies compatible with green growth.

## 5. Conclusions

Cultures of the microalgal strains *Picochlorum costavermella* VAS2.5, *Picochlorum oklahomense* SAG4.4, *Picochlorum oklahomense* PAT3.2B, *Microchloropsis gaditana* VON5.3, and *Nephroselmis pyriformis* PAT2.7 at T = 25 °C led to significant biomass production in most cases, especially taking into account that they all lasted for almost 10 days. Notably, *P. costavermella* VAS2.5, *M. gaditana* VON5.3, and *N. pyriformis* PAT2.7 grew efficiently even at T = 20 °C. Except for the genetic plasticity of these strains, the above results suggest that, in addition to CO_2_ possibly being a limiting factor, the high and lateral, which confined ‘cross-shading’, light supply in the STR played a decisive role in the increase in cell number regardless of culture temperature. The most favorable condition for lipid accumulation proved to be incubation at 25 °C for *P. costavermella* VAS2.5 and *M. gaditana* VON5.3, though *N. pyriformis* PAT2.7 increased its lipid synthesis capacity in culture under T = 20 °C. The predominant lipid fraction was glycolipids and sphingolipids (G + S) for *P. costavermella* VAS2.5 and *N. pyriformis* PAT2.7 at 25 °C and *M. gaditana* VON5.3 at 20 °C, while neutral lipids (N) were higher in the other cultures. *P. costavermella* VAS2.5 and *M. gaditana* VON5.3 proved attractive ^Δ5,8,11,14,17^C20:5 producers. Furthermore, the content in ^Δ5,8,11,14,17^C20:5 regarding *M. gaditana* VON5.3 further increased when cultured under T = 20 °C. Also, *N. pyriformis* PAT2.7 stood out as a very promising candidate for ^Δ9^C16:1 production, which was present mostly in the N fraction. On the other hand, polysaccharide content seemed to not be affected by the culture temperature, while the high protein content at low incubation temperatures (e.g., T = 20 °C) is an indicator of the microalga’s good adaptation to these conditions and suggests its suitability for large-scale outdoor cultivation where temperatures are low during the winter months. Taking into account the findings of the present research, it is evident that all strains under the proper culture conditions can be exploited on a commercial scale, mostly due to their remarkable lipid profiles and protein content, especially in the aquaculture industry. At the same time, the lipid content of *N. pyriformis* makes it an alternative candidate for the biodiesel industry.

## Figures and Tables

**Table 1:** The composition of the modified Artificial Sea Water (mASW) used as a growth medium for all cultures performed in this study.

Compound	Supplier	Concentration (g/L)
NaCl	PENTA (Prague, Czech Republic)	27.0
MgSO_4_·7H_2_O	PanReac AppliChem (Darmstadt, Germany)	6.6
CaCl_2_	PENTA	1.5
KNO_3_	Scharlau (Barcelona, Spain)	1.0
KH_2_PO_4_	Himedia (Mumbai, India)	0.07
FeCl_3_·6H_2_O	BDH (Poole, England)	0.014
Na_2_EDTA	Merck (Darmstadt, Germany)	0.019
**Microelement solution**		
ZnSO_4_·7H_2_O	Merck	40.0
H_3_BO_3_	Fluka (Steinheim, Germany)	600.0
CoCl_2_·6H_2_O	Sigma-Aldrich (St. Louis, MO, USA)	1.5
CuSO_4_·5H_2_O	BDH	40.0
MnCl_2_	Sigma-Aldrich	400.0
(NH_4_)_6_MO_7_O_24_·4H_2_O	Sigma-Aldrich	370.0

**Table 2:** Biomass production, reserve material accumulation, and growth parameters of *Picochlorum costavermella* strain VAS2.5, *Picochlorum oklahomense* SAG4.4, *P. oklahomense* PAT3.2B, *Microchloropsis gaditana* VON5.3, and *Nephroselmis pyriformis* PAT2.7 grown in mASW in a 3.7 L (V_w_ = 2.0 L) capacity Stirred Tank Reactor (STR). Culture conditions: pH = 8.5 ± 0.3, temperature: 25 ± 1 °C or 20 ± 1 °C, photoperiod: 24:0, light intensity: 1071 μmol/m^2^∙s, agitation rate: 100 rpm, aeration: 0.5 vvm. Analyses were carried out at least in duplicate.

Strain	T (°C)	Biomass (x)		Lipids (L)				Polysaccharides (S)	Proteins (P)	Pigments		Growth Parameters			
		x	*p_x_*	L/x (%)	Lipid Fractions (%)			S/x (%)	P/x (%)	TCh/x (%)	TC/x (%)	N_f_	N_max_	*μ*	R^2^
		(mg/L)	(mg/L∙d)		N	G + S	P					(cells/mL∙10^6^)		(1/d)	
*P. costavermella* VAS2.5	25	671.3 ± 17.2	67.1 ± 1.7	19.3 ± 0.7	29.6 ± 3.4	57.4 ± 5.4	13.1 ± 2.0	13.5 ± 0.8	25.0 ± 0.2	2.6 ± 0.1	0.2 ± 0.0	87.5	110.2 ±12.9	0.45 ± 0.05	0.98
	20	418.4 ± 17.7	41.8 ± 2.5	5.2 ± 0.3	UND	UND	UND	6.7 ± 0.1	26.7 ± 0.0	2.1 ± 0.2	0.8 ± 0.1	15.9	13.6 ± 2.2	1.41 ± 1.04	0.51
*P. oklahomense* SAG4.4	25	224.8 ± 5.9	22.5 ± 0.6	4.1 ± 0.4	UND	UND	UND	11.3 ± 0.4	28.8 ± 1.0	4.9 ± 0.1	0.8 ± 0.0	30.9	45.3 ± 15.6	0.39 ± 0.08	0.95
*P. oklahomense* PAT3.2B	25	242.7 ± 13.6	24.3 ± 1.4	11.5 ± 0.3	UND	UND	UND	11.2 ± 1.2	50.6 ± 3.0	6.1 ± 0.7	1.1 ± 0.1	20.6	91.8 ± 10.8	0.47 ± 0.07	0.97
*M. gaditana* VON5.3	25	527.4 ± 15.0	52.7 ± 1.5	16.4 ± 2.1	55.6 ± 1.6	24.7 ± 1.4	19.8 ± 0.3	8.5 ± 0.0	16.4 ± 2.6	1.4 ± 0.0	1.4 ± 0.0	47.7	56.7 ± 15.7	0.35 ± 0.11	0.88
	20	509.0 ± 77.8	50.9 ± 7.8	13.8 ± 0.0	22.5 ± 0.2	41.3 ± 2.0	36.3 ± 2.1	9.4 ± 2.1	17.5 ± 1.2	3.2 ± 0.3	1.1 ± 0.1	33.3	36.8 ± 6.7	0.56 ± 0.12	0.97
*N. pyriformis* PAT2.7	25	438.8 ± 89.3	43.9 ± 8.9	3.3 ± 1.7	45.8 ± 4.3	48.6 ± 5.0	6.0 ± 1.0	8.0 ± 0.2	45.2 ± 13.1	1.7 ± 0.3	0.3 ± 0.0	36.7	31.3 ± 1.3	1.47 ± 0.34	0.95
	20	546.7 ± 1.2	54.7 ± 0.1	9.3 ± 6.1	63.5 ± 6.0	34.8 ± 4.8	2.3 ± 1.2	10.7 ± 3.1	41.0 ± 10.4	1.0 ± 0.0	0.3 ± 0.0	48.8	49.3 ± 3.5	0.64 ± 0.12	0.98

Abbreviations: T (°C), temperature; x (mg/L), dry biomass; *p_x_* (mg/L∙d), volumetric biomass productivity; L/x (%), lipids on dry biomass; N (%); neutral lipid fraction on total lipids; G + S (%); glycolipids and sphingolipid fraction on total lipids; P (%), phospholipids fraction on total lipids; S/x (%), intracellular polysaccharides on dry biomass; P/x (%), intracellular proteins on dry biomass; TCh/x (%), total chlorophyll (chlorophyll-a and -b) on dry biomass; TC/x (%), total carotenoids on dry biomass; *N_f_* (cells/mL∙10^6^), number of cells at the end of culture; *N_max_* (cells/mL∙10^6^), carrying capacity of the system; *μ* (1/d), maximum specific growth rate; R^2^, R-squared statistical measure; and UND, undetermined.

**Table 3:** Fatty acid composition of total lipids (TLs) and their lipid fractions (neutral lipids—N, glycolipids—G, and phospholipids—P) of the microalgal strains that belong to the genus *Picochlorum* that were cultured in a Stirred Tank Reactor (V_w_ = 2 L) after approximately 250 h of culture. Culture conditions: modified artificial seawater (mASW), pH: 8.5 ± 0.3, temperature: 25 ± 1 °C or 20 ± 1 °C, photoperiod: 24:0, illumination: 1071 μmol/m^2^∙s, agitation: 100 rpm, aeration: 0.5 vvm. Analyses were carried out at least in duplicate.

Strain	T (°C)	Lipid Fraction	Composition of Total Lipids and Lipid Fractions in Fatty Acids (%, wt/wt)												
			C14:0	^Δ9^C14:1	C16:0	^Δ9^C16:1	C17:0	C18:0	^Δ9^C18:1	^Δ9,12^C18:2	^Δ9,12,15^C18:3	^Δ6,9,12,15^C18:4	^Δ13^C20:1	^Δ5,8,11,14,17^C20:5	* Others
*P. costavermella* VAS2.5	25	TLs	6.8 ± 0.3	3.5 ± 0.4	29.0 ± 0.2	28.6 ± 0.3	<0.5	<0.5	7.0 ± 0.6	1.1 ± 0.1	<0.5	<0.5	3.8 ± 0.1	17.0 ± 0.2	1.8 ± 0.0
		N	4.5 ± 0.2	2.8 ± 1.2	34.4 ± 2.4	29.0 ± 3.2	<0.5	1.6 ± 0.3	15.8 ± 5.7	1.4. ± 0.7	<0.5	0.8 ± 0.2	1.8 ± 0.1	5.4 ± 0.1	1.2 ± 0.3
		G	9.1 ± 0.3	4.3 ± 0.6	26.7 ± 2.2	29.6 ± 1.8	<0.5	<0.5	4.9 ± 0.0	1.1 ± 0.1	<0.5	ND	3.1 ± 0.0	18.6 ± 4.0	0.8 ± 0.4
		P	3.0 ± 0.3	0.7 ± 0.0	21.5 ± 1.0	20.1 ± 1.4	0.7 ± 0.1	<0.5	10.1 ± 0.1	3.2 ± 1.3	1.3 ± 0.1	ND	7.9 ± 1.0	30.3 ± 4.9	1.0 ± 0.2
	20	TLs	6.3 ± 0.5	4.1 ± 0.1	14.3 ± 0.6	26.8 ± 0.9	<0.5	<0.5	4.7 ± 0.3	1.4 ± 0.3	ND	ND	3.7 ± 0.7	35.9 ± 0.7	2.3 ± 0.1
*P. oklahomense* SAG4.4	25	TLs	2.4 ± 0.2	8.5 ± 0.5	12.2 ± 2.1	4.7 ± 0.6	3.7 ± 0.2	7.2 ± 0.0	18.4 ± 2.4	8.2 ± 0.2	6.3 ± 0.6	18.9 ± 3.6	ND	ND	9.5 ± 0.3
*P. oklahomense* PAT3.2B	25	TLs	2.3 ± 0.2	9.3 ± 0.3	15.2 ± 0.1	4.4 ± 0.4	ND	6.4 ± 0.7	14.4 ± 0.8	25.3 ± 0.0	17.9 ± 1.0	ND	ND	ND	4.8 ± 0.0

Abbreviations: ND: not detected. * Others: mainly C10:0, C12:0, and in some cases, ^Δ6,9,12^C18:3. Note: only glycolipids (i.e., G fraction) are mentioned as the amide bond of sphingolipids resists methanolysis during methyl esterification.

**Table 4:** Fatty acid composition of total lipids (TLs) and their lipid fractions (neutral lipids—N, glycolipids—G, and phospholipids—P) of the microalgal strain *Microchloropsis gaditana* VON5.3 that was cultured in a Stirred Tank Reactor (V_w_ = 2 L) after approximately 250 h of culture. Culture conditions: modified artificial seawater (mASW), pH: 8.5 ± 0.3, temperature: 25 ± 1 °C or 20 ± 1 °C, photoperiod: 24:0, illumination: 1071 μmol/m^2^∙s, agitation: 100 rpm, aeration: 0.5 vvm. Analyses were carried out at least in duplicate.

Strain	T (°C)	Lipid Fraction	Composition of Total Lipids and Lipid Fractions in Fatty Acids (%, wt/wt)												
			C14:0	^Δ9^C14:1	C16:0	^Δ9^C16:1	C17:0	C18:0	^Δ9^C18:1	^Δ9,12^C18:2	^Δ9,12,15^C18:3	^Δ6,9,12,15^C18:4	^Δ13^C20:1	^Δ5,8,11,14,17^C20:5	* Others
*Microchloropsis gaditana* VON5.3	25	TLs	7.2 ± 0.6	3.7 ± 0.7	24.1 ± 1.3	30.5 ± 0.8	<0.5	<0.5	5.5 ± 1.3	2.0 ± 0.0	<0.5	ND	3.4 ± 0.5	19.9 ± 0.1	2.9 ± 0.7
		N	6.6 ± 0.9	3.5 ± 0.5	27.5 ± 1.1	34.3 ± 0.6	0.6 ± 0.1	0.7 ± 0.1	6.7 ± 1.4	1.7 ± 0.3	<0.5	ND	3.1 ± 0.6	10.4 ± 1.9	2.1 ± 0.2
		G	10.1 ± 1.0	5.3 ± 0.3	22.6 ± 1.8	28.7 ± 0.6	<0.5	<0.5	5.0 ± 1.4	2.0 ± 0.7	0.6 ± 0.0	ND	2.5 ± 0.5	19.6 ± 2.7	2.4 ± 0.1
		P	2.8 ± 0.0	1.7 ± 0.1	36.3 ± 1.0	8.4 ± 0.1	<0.5	17.6 ± 0.9	13.0 ± 0.6	1.6 ± 0.6	0.6 ± 0.0	ND	5.9 ± 1.4	10.9 ± 0.2	1.1 ± 0.1
	20	TLs	7.0 ± 0.5	3.4 ± 0.5	27.1 ± 1.7	30.4 ± 0.3	ND	1.0 ± 0.4	6.0 ± 2.8	1.8 ± 0.5	2.2 ± 1.4	0.6 ± 0.0	2.8 ± 0.9	16.0 ± 6.9	1.8 ± 0.1
		N	5.5 ± 0.3	2.5 ± 1.3	32.4 ± 1.2	37.8 ± 1.5	<0.5	0.9 ± 0.1	5.9 ± 0.4	1.1 ± 0.1	0.7 ± 0.0	1.5 ± 0.6	2.0 ± 0.0	8.2 ± 0.1	0.9 ± 0.2
		G	9.0 ± 0.2	5.6 ± 0.8	24.3 ± 0.7	28.1 ± 0.7	<0.5	<0.5	2.4 ± 0.3	1.2 ± 0.0	<0.5	<0.5	1.4 ± 0.1	18.7 ± 2.3	2.2 ± 0.2
		P	2.6 ± 0.0	1.1 ± 0.1	17.6 ± 0.8	28.9 ± 1.7	<0.5	<0.5	5.1 ± 0.2	2.6 ± 0.0	1.0 ± 0.1	ND	7.5 ± 1.0	29.5 ± 3.3	0.8 ± 0.1

Abbreviations: ND: not detected. * Others: mainly C10:0, C12:0, and in some cases, ^Δ6,9,12^C18:3. Note: only glycolipids (i.e., G fraction) are mentioned as the amide bond of sphingolipids resists methanolysis during methyl esterification.

**Table 5:** Fatty acid composition of total lipids (TLs) and their lipid fractions (neutral lipids—N, glycolipids—G, and phospholipids—P) of the microalgal strain *Nephroselmis pyriformis* PAT2.7 that was cultured in a Stirred Tank Reactor (V_w_ = 2 L) after approximately 250 h of culture. Culture conditions: modified artificial seawater (mASW), pH: 8.5 ± 0.3, temperature: 25 ± 1 °C or 20 ± 1 °C, photoperiod: 24:0, illumination: 1071 μmol/m^2^∙s, agitation: 100 rpm, aeration: 0.5 vvm. Analyses were carried out at least in duplicate.

Strain	T (°C)	Lipid Fraction	Composition of Total Lipids and Lipid Fractions in Fatty Acids (%, wt/wt)							
			C14:0	^Δ9^C14:1	C16:0	^Δ9^C16:1	C18:0	^Δ9^C18:1	^Δ9,12^C18:2	* Others
*Nephroselmis pyriformis* PAT2.7	25	TLs	31.0 ± 0.8	7.2 ± 1.2	9.2 ± 0.6	40.1 ± 0.1	2.0 ± 0.7	3.7 ± 2.0	2.2 ± 0.3	4.8 ± 3.2
		N	36.0 ± 1.4	6.0 ± 0.3	9.4 ± 0.6	43.0 ± 1.9	0.7 ± 0.3	1.6 ± 0.8	1.4 ± 0.0	1.6 ± 0.5
		G	31.9 ± 0.2	5.4 ± 0.7	8.2 ± 0.4	39.8 ± 0.4	2.2 ± 1.1	3.2 ± 0.7	<0.5	8.8 ± 1.9
		P	15.5 ± 0.7	4.4 ± 3.9	14.3 ± 0.6	29.8 ± 0.2	5.8 ± 1.1	19.4 ± 2.3	1.9 ± 0.5	9.1 ± 0.1
	20	TLs	30.8 ± 1.0	5.9 ± 0.1	8.4 ± 0.2	39.2 ± 0.9	1.2 ± 0.1	1.4 ± 0.3	ND	13.1 ± 1.2
		N	37.4 ± 1.8	6.3 ± 0.2	8.8 ± 0.8	44.8 ± 0.9	<0.5	0.8 ± 0.0	ND	0.9 ± 0.2
		G	30.3 ± 0.4	17.3 ± 0.0	7.6 ± 0.4	22.1 ± 0.0	2.5 ± 0.1	4.7 ± 0.5	ND	7.8 ± 1.2
		P	20.3 ± 3.4	1.5 ± 0.2	14.5 ± 0.5	15.1 ± 1.3	8.7 ± 0.2	20.2 ± 5.7	ND	10.5 ± 0.1

Abbreviations: ND: not detected. * Others: mainly C10:0, C12:0, and in some cases ^Δ6,9,12^C18:3. Note: only glycolipids (i.e., G fraction) are mentioned as the amide bond of sphingolipids resists methanolysis during methyl esterification.

## Data Availability

The original contributions presented in this study are included in the article. Further inquiries can be directed to the corresponding author.

## Abbreviations

The following abbreviations are used in this manuscript:

PUFAs	polyunsaturated fatty acids
ALA	α-linolenic acid
EPA	Eicosapentaenoic acid
DHA	Docosahexaenoic acid
STR	Stirred Tank Reactor
mASW	modified Artificial Sea Water
